# MMVFL: A Simple Vertical Federated Learning Framework for Multi-Class Multi-Participant Scenarios

**DOI:** 10.3390/s24020619

**Published:** 2024-01-18

**Authors:** Siwei Feng, Han Yu, Yuebing Zhu

**Affiliations:** 1 School of Computer Science & Technology, Soochow University, Suzhou 215000, China; 20215227123@stu.suda.edu.cn; 2 School of Computer Science and Engineering, Nanyang Technological University, Singapore 639798, Singapore; han.yu@ntu.edu.sg

**Keywords:** vertical federated learning, multiple participants, multiple classes, multi-view learning

## Abstract

Federated learning (FL) is a privacy-preserving collective machine learning paradigm. Vertical federated learning (VFL) deals with the case where participants share the same sample ID space but have different feature spaces, while label information is owned by one participant. Early studies of VFL supported two participants and focused on binary-class logistic regression problems, while recent studies have put more attention on specific aspects such as communication efficiency and data security. In this paper, we propose the multi-participant multi-class vertical federated learning (MMVFL) framework for multi-class VFL problems involving multiple parties. By extending the idea of multi-view learning (MVL), MMVFL enables label sharing from its owner to other VFL participants in a privacy-preserving manner. To demonstrate the effectiveness of MMVFL, a feature selection scheme is incorporated into MMVFL to compare its performance against supervised feature selection and MVL-based approaches. The proposed framework is capable of quantifying feature importance and measuring participant contributions. It is also simple and easy to combine with other communication and security techniques. The experiment results on feature selection for classification tasks on real-world datasets show that MMVFL can effectively share label information among multiple VFL participants and match the multi-class classification performance of existing approaches.

## 1. Introduction

Traditional machine learning approaches require that all data and learning processes gather in a central entity. This limits their ability to deal with real-world applications where data are isolated across different organizations and data privacy is emphasized. Federated learning (FL) [1,2,3,4,5,6] is a distributed machine learning paradigm for learning from distributed data silos without the need to expose private information, which has been widely applied in many areas such as healthcare [7], finance [8], autonomous driving [9], and recommendation [10]. FL integrates several powerful machine learning techniques, such as deep learning, reinforcement learning, transfer learning, and ensemble learning, to enhance its capabilities and performance. It is well suited for such scenarios and has attracted growing attention. While FL has been studied in different tasks (e.g., classification [11] or regression [12]), we focus in this paper on classification.

**Figure 1:**
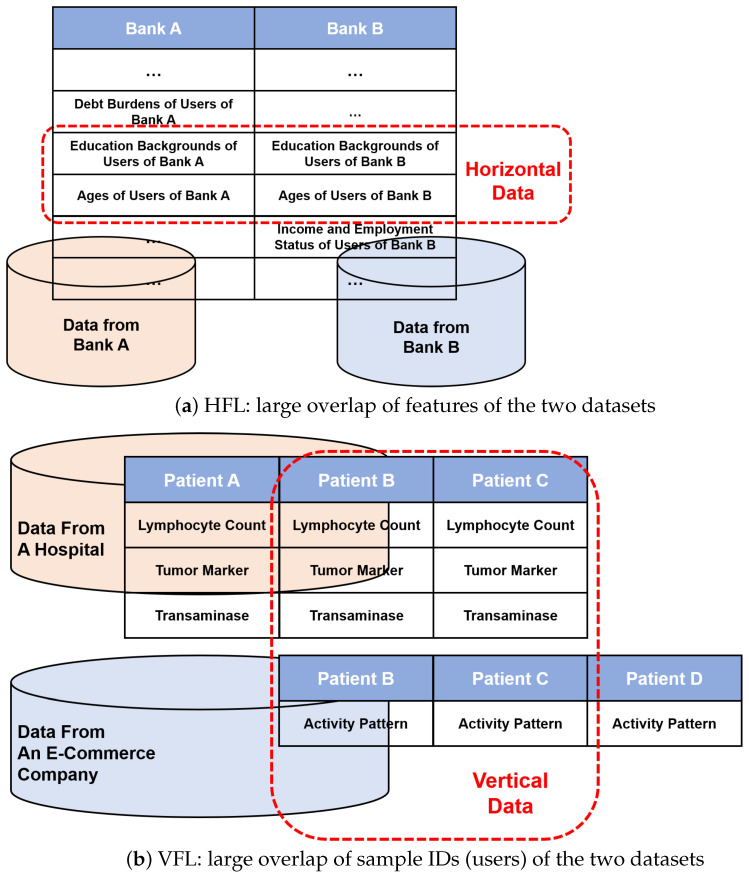
HFL vs. VFL.

Existing FL approaches mostly focus on horizontal scenarios [13,14,15,16] and assume that datasets from different participants share the same feature space but may not share the same sample ID space (Figure 1, top). Most existing horizontal federated learning (HFL) approaches aim to train a single global model for all participants [17,18], while a few focus on learning separate models for each participant [11]. However, this is not always true in practice. For example, a hospital, which operates solely in one city, is devising a plan to conduct feature selection using patients’ data for disease prediction. The aim is to identify the crucial factors that contribute to disease prediction. However, the existing feature dimensions related to patients’ physical conditions, such as blood pressure, breath sounds, etc., lack sufficient valuable information that can be utilized to learn insightful features. In such a scenario, if another organization, such as a wearable healthcare device company that operates in the same city, shares its data with the hospital, it could potentially provide additional feature dimensions related to patients’ physical conditions from the sensor data, such as activity pattern. The rationale behind this is that it is natural to assume that overlap exists between the user groups of these two organizations because they have business in the same city. The inclusion of these supplementary dimensions may assist the hospital in identifying the most influential factors for accurate disease prediction. In this case, data from different parties may share little overlap in feature space, thereby rendering the use of HFL schemes impractical.

Vertical federated learning (VFL) [19,20,21,22,23,24,25,26,27,28] assumes that datasets from different participants do not share the same feature space but may share the same sample ID space (Figure 1, bottom). Furthermore, label information is assumed to be held by one participant. Therefore, in the example mentioned earlier, the two organizations can adopt a VFL scheme, enabling the hospital to leverage the wearable healthcare device company’s users’ sensor data without the need for direct sharing of raw data. This can be achieved by implementing the VFL methodology. In this case, both organizations possess patients’ data with different feature dimensions, but only the hospital who acts as the task curator holds label information (e.g., personal loan default information) of users of both organizations for the intended feature selection task. Though having promising potential in many applications with privacy preservation, VFL is currently less explored than HFL [29] because current VFL methods are unable to handle real-world applications effectively due to their design limitations. To be more concrete, the early studies on VFL primarily focused on developing VFL frameworks and addressing challenges related to entity resolution errors, among which Hardy et al. [19] proposed a federated logistic regression scheme with encrypted messages, while Nock et al. [20] extended it to evaluate the impact of entity resolution errors across different losses. Yang et al. [21] and Yang et al. [22] introduced variations of [19] assuming prematched sample IDs, aiming to reduce communication rounds and enhance privacy. Wang et al. [30] proposed a method to evaluate feature importance in VFL participants’ local datasets using Shapley values [31]. Each of these VFL schemes can handle only two VFL participants and are generally focused on binary classification tasks [19,20,21,22,30]. This makes them unsuitable for complex classification tasks in VFL applications involving multiple participants. Though recent studies have partially alleviated these limitations, they either focus on certain aspects of VFL such as alleviating information leakage and defending attacks [25,27,32,33,34,35,36] and improving communication efficiency in an asynchronous manner [24,37], or are designed for specific applications with additional information required [26,38]. Therefore, there is a need for a general VFL framework that can effectively address intricate classification tasks in scenarios where multiple participants are involved.

In this paper, we propose the multi-participant multi-class vertical federated learning (MMVFL) framework. It extends the idea of multi-view learning (MVL) [39], which jointly learns multiple models for tasks of multiple separate views of the same input data, to establish a VFL framework that is suitable for multi-class problems with multiple participants. Though most existing studies on FL tend to build a global model, it is widely recognized that the primary objective of federated learning is to improve model performance at each client through collaborative learning while ensuring data privacy. While building a global model is one approach to achieve this goal, it is not the sole method. In this paper, like the multi-task FL framework proposed in [11], MMVFL learns a separate model for each participant, instead of a single global model for all participants, to make the learning process more personalized. Furthermore, MMVFL enables label sharing from the label owner to other participants to facilitate federated model training. It is worth mentioning that MMVFL is privacy-preserving, which means data and labels do not leave their owners during the training process. In addition, we propose a feature importance evaluation scheme based on MMVFL. It can quantify the contribution of different features from each participant to the FL model. By discarding redundant and harmful features in initial training periods, the communication, computation, and storage costs of a VFL system can be reduced for subsequent training under incremental learning settings. To the best of our knowledge, MMVFL is the first VFL framework to be used for a multi-class problem with multiple participants. Through extensive experimental evaluation, we demonstrate that MMVFL can effectively share label information among multiple VFL participants and match the multi-class classification performance of the existing approaches.

The contributions of the proposed method are summarized as follows:

- A novel and simple multi-participant multi-class VFL framework is proposed. By using this framework, a data owner can borrow information from other data owners to help improve task performance without raw data being disclosed.
- A feature importance evaluation scheme based on MMVFL is proposed, which aims to assess the significance of different features contributed by each participant in the federated learning (FL) model. By identifying and eliminating redundant and harmful features during the initial training stages, the subsequent learning process can be conducted with reduced communication, computation, and storage costs.
- The proposed framework is flexible because different schemes that aim at enhancing communication efficiency and security can be incorporated into the framework. In addition, MMVFL can be easily extended by combining it with deep neural networks.
- The proposed MMVFL framework has been evaluated extensively based on two datasets. The results show that MMVFL is comparable with, and in some cases even superior to, methods that provide label information to each client.

## 2. Related Work

### 2.1. Vertical Federated Learning

VFL is suitable for FL scenarios in which participants have datasets that share the same sample ID space but have a different feature space. Early studies on VFL focused on the building of VFL frameworks. The idea of VFL was first proposed in [19], where a federated logistic regression scheme was designed with messages encrypted with an additively homomorphic scheme. It also provided a formal analysis of the impact of entity resolution mistakes on learning. Reference [20] then extended [19] to provide a formal assessment of the impact of errors in entity resolution on learning that spans a wide set of losses. Refs. [21,22] are two extensions of [19] that assume sample IDs being already matched. The former focused on reducing the rounds of communication required by proposing a limited-memory Broyden–Fletcher–Goldfarb–Shanno algorithm [40] based on a privacy-preserving optimization framework. The latter built a parallel distributed system by removing the third-party coordinator to decrease the risk of data leakage and reduce the complexity of the system. In [30], the authors proposed an approach to evaluate feature importance in VFL participants’ local dataset. The approach dynamically removes different groups of features to assess the impact on FL model performance following a Shapley-value-based method. It is able to evaluate feature importance at the granularity of feature groups. In addition, the computation of Shapley values incurs exponential computational complexity, making it hard to scale up. Nevertheless, these approaches are only able to deal with two VFL participants, and are generally focused on binary classification tasks. This limits the applicability of these methods in real-world application scenarios.

Though recent studies in VFL have alleviated the limitations of early VFL frameworks to a certain degree, they either focus on certain aspects of VFL (e.g., security, communication efficiency, etc.) or are designed for specific applications with additional information required. For example, Refs. [25,27,32,33,34,35,36] focus on alleviating information leakage and defending attacks in VFL. References [24,37] present approaches to enhancing communication efficiency in an asynchronous manner. References [26,38] propose VFL methods that rely on deep neural networks, with non-overlapping samples needed for performance improvement. Moreover, Ref. [26] designs methods specifically for feature selection.

### 2.2. Embedded Feature Selection

The fundamental concept behind embedded feature selection involves utilizing a transformation matrix to project data onto a new space. The selection of features is then guided by the sparsity of the transformation matrix. This principle forms the basis for the general framework of embedded feature selection methods, which can be expressed through the following optimization process:

$$\min_{W}L\left(Y,\mathrm{WX}\right)+\lambda R\left(W\right),$$

where Y denotes the label matrix for supervised settings, $L\left(\cdot \right)$ denotes a loss function, and $R\left(\cdot \right)$ denotes a regularization function to enforce sparsity on the transformation matrix W, which further guide feature selection. One basic assumption of most existing embedded feature selection methods [41,42,43,44] is that the data to be processed lie in or near a completely linear low-dimensional manifold, but this is not always true in practice. To tackle this problem, Feng et al. [45] proposed the use of an autoencoder instead of a transformation matrix to perform data projection. The non-linear nature of the model and broad goal of data reconstruction enable the autoencoder to provide a more generalized (or non-linear) embedding that captures the manifold structure of the input data.

Most existing embedded feature selection algorithms primarily focus on scenarios where the data originate from a single source. However, to fully harness the benefits of multiple data sources, collaborative multi-source feature selection algorithms have been developed [46,47,48]. These methods aim to leverage information from different sources in a collective manner. However, a significant drawback of these approaches is that they necessitate the sharing of data among the parties involved, rendering them unsuitable for situations where data security and privacy are paramount. In response to this challenge, Ye et al. [49] proposed an alternating approach that facilitates feature selection in a collaborative manner while preserving data security. Their method involves utilizing intermediate representations of data at each party, preventing the disclosure of sensitive local information. However, it should be noted that this approach has certain limitations. Firstly, it only employs overlapping samples for training, which may restrict its effectiveness in scenarios with limited sample overlap. Additionally, it assumes that the data from different parties possess identical dimensionality, which can severely constrain its practical applicability.

### 2.3. Multi-View Learning

MVL approaches aim to learn one function to model each view and jointly optimize all the functions to improve generalization performance [39]. Data from each view are assumed to share the same sample ID space but with heterogeneous features, making MVL well-suited for the VFL scenario. Unfortunately, existing MVL methods require raw data from different views to interact during learning, making them not suitable for direct application in FL due to them violating the privacy preservation requirement.

## 3. Proposed Method

**Figure 2:**
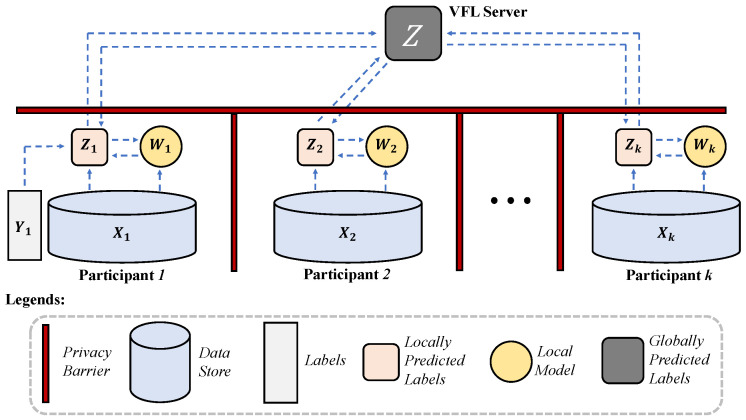
The pipeline of MMVFL.

The pipeline of MMVFL is shown in Figure 2. First, local models are learned independently at each party to obtain predictions for sharing. After that, predictions from all parties are sent to the server to generate a global prediction for the next round of local training. The learning process is performed in an end-to-end manner, with classification errors being minimized along with a sparse regularization term acting on the transformation matrix at each party for feature importance characterization. Feature selection is then performed based on the obtained feature importance. By design, only the locally predicted labels cross the privacy barriers to reach the VFL server. The server operates without raw data, labels, or local models leaving their owners’ machine. In this section, we present the problem definition and the details of MMVFL.

### 3.1. Notations and Problem Definition

Throughout this paper, matrices are denoted as bold upper-case letters. For a matrix $A\in R^{R\times C}$, ${∥A∥}_{2,1}=\sum_{i=1}^{R}{∥A^{\left(i\right)}∥}_{2}$ denotes the $l_{2,1}$-norm of A, where $∥A^{\left(i\right)}{∥}_{2}$ denotes the vector corresponding to the *i*th row of A.

For a VFL task for a $N_{c}$-class problem involving *K* participants, each participant owns a dataset $X_{k}\in R^{N\times d_{k}}$ stored locally for FL model training. $d_{k}$ denotes the dimensionality of the dataset and *N* denotes the number of samples in it. Following the setup in [19], label information is assumed to be owned by one participant. Without loss of generality, we assume that the first participant owns the labels. The research problem here is how to transfer label information from the first participant to others for VFL model training while performing a feature importance evaluation for each participant. We assume that sample IDs are already matched in this paper. Notations used in this paper are listed in Table 1.

### 3.2. Sparse-Learning-Based Unsupervised Feature Selection

For participants who do not have access to the label information, unsupervised feature selection is adopted to select features that are representative of the underlying subspace structure of the data [50]. A transformation matrix is designed to project data to a new space and guide feature selection based on the sparsity of the transformation matrix.

MMVFL performs feature selection on the *k*th participant by optimizing the following objective function:

$$\begin{array}{cc} \; & \min_{W_{k},Z_{k}}∥X_{k}W_{k}-Z_{k}{∥}_{F}^{2}+\beta_{k}{∥W_{k}∥}_{2,1}\\ \; & s.t.\;Z_{k}^{T}Z_{k}=I,\;Z_{k}⩾0 \end{array}$$

where $\beta_{k}$ is a balance parameter, $W_{k}\in R^{d_{k}\times N_{c}}$ is the transformation matrix, and $Z_{k}\in R^{N\times N_{c}}$ is an embedding matrix in which each row denotes the representation of the corresponding data point. The second term is used as a regularization function to enhance the feature importance measure. The two constraints enable $Z_{k}$ to serve as a pseudo-label matrix for $X_{k}$.

Once $W_{k}$ is produced, a feature importance score for each feature is computed by the $l_{2}$-norm value of the corresponding row of $W_{k}$ following [51]. Although sophisticated sparse-learning-based unsupervised feature selection algorithms have been proposed in recent years, we adopt the linear transformation method for its simplicity as our focus is to provide a proof-of-concept rather than exhausting all possible feature selection schemes.

Filter-based feature selection is then performed independently on each client using the score function $S_{k}\left(i\right)={∥{W_{k}}_{\left(i\right)}∥}_{2}$, where $i=1,\;2,\;\cdots ,\;d_{k}$ denotes the *i*th feature of data in client *k*. Features with higher scores are given higher priority for selection.

### 3.3. Privacy-Preserving Label Sharing

Since most MVL approaches assume that all views share the same label space and they are correlated through the label space, following [52], the local feature selection scheme in Section 3.2 can be adapted to MVL as follows:

(1) $$\begin{array}{cc} \; & \min_{W_{k},Z}\;\sum_{k=1}^{K}∥X_{k}W_{k}{-Z∥}_{F}^{2}+\beta_{k}{∥W_{k}∥}_{2,1}\\ \; & s.t.\;Z^{T}Z=I,\;Z⩾0. \end{array}$$

However, the optimization of Z needs access to raw data from different views. Thus, it cannot be directly applied to VFL. To adapt Equation (1) to VFL, we propose the following objective function:

(2) $$\begin{array}{cc} \; & \min_{W_{k},Z_{k},Z}\;\sum_{k=1}^{K}∥X_{k}W_{k}-Z_{k}{∥}_{F}^{2}+\beta_{k}{∥W_{k}∥}_{2,1}\\ \; & s.t.\;Z_{1}=Y,\;Z_{k}=Z,\;Z_{k}⩾0,\;Z_{k}^{T}Z_{k}=I \end{array}$$

where $Y\in {\left\{0,1\right\}}^{N\times Nc}$ is a one-hot matrix containing the label information that is owned by the first participant.

Following Equation (2), each participant trains a pseudo-label matrix $Z_{k}$ locally. The constraint condition $Z_{k}=Z$ ensures that these locally learned matrices are equal (Z is an implementation that data from all participants share the same label space). The constraint condition $Z_{1}=Y$ ensures that the pseudo-labels learned by the first participant are equal to the true labels. Note that the combination of the two constraint conditions $Z_{k}=Z$ and $Z_{1}=Y$ indirectly ensures that $Z_{k}$ is equal to Y. This achieves label sharing without direct access to raw data from different participants, making it suitable for VFL operations.

### 3.4. Optimization

Following [46], we relax the constraints of $Z_{k}=Z$ and $Z_{1}=Y$ by adding a large enough penalty term $\zeta_{k}$ and η to each of them, respectively. Equation (2) can be rewritten as:

(3) $$\begin{array}{cc} \min_{W_{k},Z_{k},Z}\; & \sum_{k=1}^{K}∥X_{k}W_{k}-Z_{k}{∥}_{F}^{2}+\beta_{k}{∥W_{k}∥}_{2,1}+\\ \; & \zeta_{k}∥Z_{k}{-Z∥}_{F}^{2}+\eta {∥Z_{1}-Y∥}_{F}^{2} \end{array}$$

Note that the constraints $Z_{k}^{T}Z_{k}=I$ and $Z_{k}⩾0$ are ignored because the large values of $\zeta_{k}$ and η ensure that $Z_{k}$ is close to Y. The fact that Y satisfies $Y^{T}Y=I$ and Y⩾0 makes the two constraints redundant.

The closed-form solution of the optimization problem in Equation (3) is hard to obtain due to the $l_{2,1}$-norm regularization term. To solve it, we design an alternating optimization approach with all parameters being iteratively updated, until the objective function value in Equation (3) converges or a maximum number of iterations is reached. That is:

- When $Z_{k}$ and Z are fixed, $W_{k}$ can be solved locally. Equation (3) becomes:
  $$\min_{W_{k}}∥X_{k}W_{k}-Z_{k}{∥}_{F}^{2}+\beta_{k}{∥W_{k}∥}_{2,1}.$$
  Though $∥W_{k}{∥}_{2,1}$ is convex, its derivative does not exist when $W_{k}\left(i\right)=0$. Following [43], by denoting $F\left(W_{k}\right)=∥X_{k}W_{k}-Z_{k}{∥}_{F}^{2}+\beta_{k}{∥W_{k}∥}_{2,1}$, its derivative with respect to $W_{k}$ is
  $$\frac{\partial F\left(W_{k}\right)}{\partial W_{k}}=2{X_{k}}^{T}X_{k}W_{k}-2{Z_{k}}^{T}X_{k}+2\beta_{k}A_{k}W_{k}$$
  where $A_{k}\in R^{d_{k}\times d_{k}}$ is a diagonal matrix whose *i*th element on the diagonal is
  (4) $$A_{k}^{\left(i,i\right)}=1/[2\left(∥W_{k}{\left(i\right)∥}_{2}+\epsilon \right))].$$
  ϵ is a small constant to avoid overflow. Thus, $∥W_{k}{\left(i\right)∥}_{2}$ is nonzero for every *i*. Therefore, Equation (4) can be rewritten as:
  (5) $$\min_{W_{k},A_{k}}{∥X_{k}W_{k}-Z_{k}∥}_{F}^{2}+\beta_{k}\mathrm{Tr}\left(W_{k}^{T}A_{k}W_{k}\right)$$
  We employ an alternating optimization scheme to solve Equation (5). When $A_{k}$ is fixed, the optimal value of $W_{k}$ can be obtained through
  (6) $$W_{k}^{*}={\left(X_{k}^{T}X_{k}+\beta A_{k}\right)}^{-1}X_{k}^{T}Z_{k}.$$
  When $W_{k}$ is fixed, we can update $A_{k}$ through Equation (4). Note that $W_{k}$ is initialized with random values before the optimization process begins.
- When $W_{k}$ is fixed, the optimization problem for solving $Z_{k}$ and Z is
  (7) $$\min_{Z_{k},Z}\;\sum_{k=1}^{K}∥X_{k}W_{k}-Z_{k}{∥}_{F}^{2}+\zeta_{k}∥Z_{k}{-Z∥}_{F}^{2}+\eta_{1}{∥Z_{1}-Y∥}_{F}^{2}$$
  The optimization of Equation (7) is performed in an alternating manner. When $Z_{k}$, k=2, 3, ⋯, K and Z are fixed, $Z_{1}$ can be solved locally through
  $$\begin{array}{c} \min_{Z_{1}}\;∥X_{1}W_{1}-Z_{1}{∥}_{F}^{2}+\zeta_{1}∥Z_{1}{-Z∥}_{F}^{2}+\eta_{1}{∥Z_{1}-Y∥}_{F}^{2} \end{array}$$
  It is straightforward to obtain the optimal $Z_{1}$ by simply taking the derivative as
  $$Z_{1}^{*}=\left(X_{1}W_{1}+\zeta_{1}Z+\eta Y\right)/\left(1+\zeta_{1}+\eta \right)$$
  When $Z_{1}$ and Z are fixed, the optimization of $Z_{k}$ for k=2, 3, ⋯, K can be carried out in a similar way, and the optimal $Z_{k}$ is:
  $$Z_{k}^{*}=\left(X_{k}W_{k}+\zeta_{k}Z\right)/\left(1+\zeta_{k}\right)$$
  Likewise, when ${\left\{Z_{k}\right\}}_{k=1}^{K}$ are fixed, the optimal value of Z is:
  (8) $$Z^{*}=\frac{\sum_{k=1}^{K}\zeta_{k}Z_{k}}{\sum_{k=1}^{K}\zeta_{k}}$$

## 4. Analysis

### 4.1. Convergence

The optimization problems for $Z_{1}$, $Z_{k}$ (k=1, 2, ⋯, K), and Z, when other parameters are fixed, are all simple convex optimization problems with global minima. It can be easily shown that the optimization scheme for $W_{k}$ is able to make Equation (5) consistently decrease until convergence following the same analysis in [43]. Interested readers can refer to [43] for details. In this way, the objective function is consistently non-increasing during optimization.

### 4.2. Time Complexity

For the *k*th participant in VFL, the most time-consuming part during local training under MMVFL is the optimization of $W_{k}$ following Equation (6). The time complexity is $O(d_{k}^{3})$. Since the proposed optimization scheme requires per-iteration communications among all participants, the time complexity of each iteration of the federated learning is $O({\left(\max_{k}\left(d_{k}\right)\right)}^{3})$, which means the time taken for FL training under MMVFL depends on the slowest participant in each round (referred to as stragglers). Techniques such as those reported in [53] can be used to improve the communication efficiency. We do not delve into the details of such techniques here.

### 4.3. Privacy Preservation

The main idea of MMVFL is that each participant learns its own model parameters $W_{k}$ and $Z_{k}$ locally, while Z is updated in a federated manner as expressed in Equation (8). In this process, only $Z_{k}$ values from all participants are required to be transmitted to the FL server, while $X_{k}$ and Y values are stored locally by their owners. Therefore, MMVFL provides a privacy-preserving label sharing as the transformation matrices are not enough to be used to derive the original data even when they are intercepted by a malicious entity in multiple rounds. In this paper, the design of MMVFL meets the minimum requirements of federated learning (i.e., no data and label sharing) instead of integrating more sophisticated data security protection schemes as our focus is to provide a proof-of-concept. However, note that MMVFL can be easily combined with these schemes to enhance robustness.

## 5. Experimental Evaluation

In this section, we evaluate the performance of MMVFL in terms of its effectiveness in label sharing. Experiments are conducted on two benchmark computer vision datasets.

### 5.1. Dataset Information

We perform experiments on 4 benchmark MVL datasets, including 2 image datasets (Handwritten and Caltech7 [54]), 1 text dataset (Relathe), and 1 audio dataset (Isolet). Both Handwritten and Caltech7 contain 6 views. However, for Handwritten, we remove the view with morphological features because it only contains 6 features, which makes feature selection insignificant. As a result, in our experiment, Handwritten has 5 views and Caltech7 has 6 views, which can be regarded as coming from 5 and 6 VFL participants with each owning data with features from one view, respectively. For both Relathe and Isolet, we split the datasets along the feature dimension into 3 parts to analog the scenario involving 3 participants. In order to eliminate the side effect caused by imbalanced classes, for each dataset, we ensure the number of instances from each class are the same for both the training and the validation sets. The properties of the datasets in our experiments are summarized in Table 2.

### 5.2. Comparison Baselines

MMVFL is the first general VFL framework capable of effectively tackling complex classification tasks in scenarios involving multiple participants. Consequently, none of the studies introduced in Section 1 and Section 2 are suitable for direct comparison as they either possess design limitations (restricted to binary classification tasks or scenarios with binary participants) or focus on other applications such as security and communication efficiency rather than classification. In order to evaluate the performance of MMVFL in collaborative learning, the following two relevant methods that grant each client direct access to label information are selected for comparison:

1. supFL [41]: which performs independent supervised feature selection on each of the *K* participants assuming that they all have access to label information. It optimizes the following objective function:
  (9) $$\min_{W_{k}}∥X_{k}W_{k}{-Y∥}_{F}^{2}+\beta_{k}{∥W_{k}∥}_{2,1}.$$
  Note that notation Y in Equation (9) refers to the one-hot matrix that contains the label information as defined in Section 3.3, which is different from the same notation used in [41].
2. supMVLFL: which performs supervised multi-view feature selection under a linear transformation framework. It is a direct extension of supFL [41] into an MVL architecture, which optimizes the following objective function:
  (10) $$\min_{W_{k}}\sum_{k=1}^{K}∥X_{k}W_{k}{-Y∥}_{F}^{2}+\beta_{k}{∥W_{k}∥}_{2,1}.$$

According to [52], MVL can improve learning performance for each view compared to learning separately as multiple views can complement each other and and reduce the effect of noisy and partial data for separate single-view learning put together. The above two approaches are distributed machine learning approaches capable of sharing information across multiple participants but do not preserve data privacy in this process.

### 5.3. Experiment Settings

We fixed some parameters and tuned others according to a “grid search” strategy. For all algorithms, we set the balance parameters $\beta_{k}=\beta$ and $\zeta_{k}=\zeta$, ∀k for simplicity, where $\beta \in \{10^{-5},10^{-4},10^{-3},10^{-2},10^{-1},1,10\}$ and ζ=1000. We also set $\eta_{1}=1000$.

We performed a 5-fold cross-validation for classification. That is, for each view on a given dataset, samples from each class are divided equally into 5 parts. Five training/validation processes are conducted separately. Four out of the five parts are used together as the training set, while the remaining part is used as the validation set. For each specific fold and each specific view on a given dataset, after the transformation matrix is obtained for each participant, we first perform feature importance evaluation based on the scheme proposed in Section 3.2. Then, we keep the top p% of the features with the highest importance during validation. We select p∈{2,4,6,8,10,20,30,40,50,60,70,80,90,100} of all the features from each dataset. For each specific value of *p*, each specific fold, and each specific view on a certain dataset, we tune the parameters for each algorithm in order to achieve the best results among all possible parameter combinations. Finally, we report the averaged classification accuracy of 5-fold cross-validation for each view of each dataset.

### 5.4. Results and Discussion

**Figure 3:**
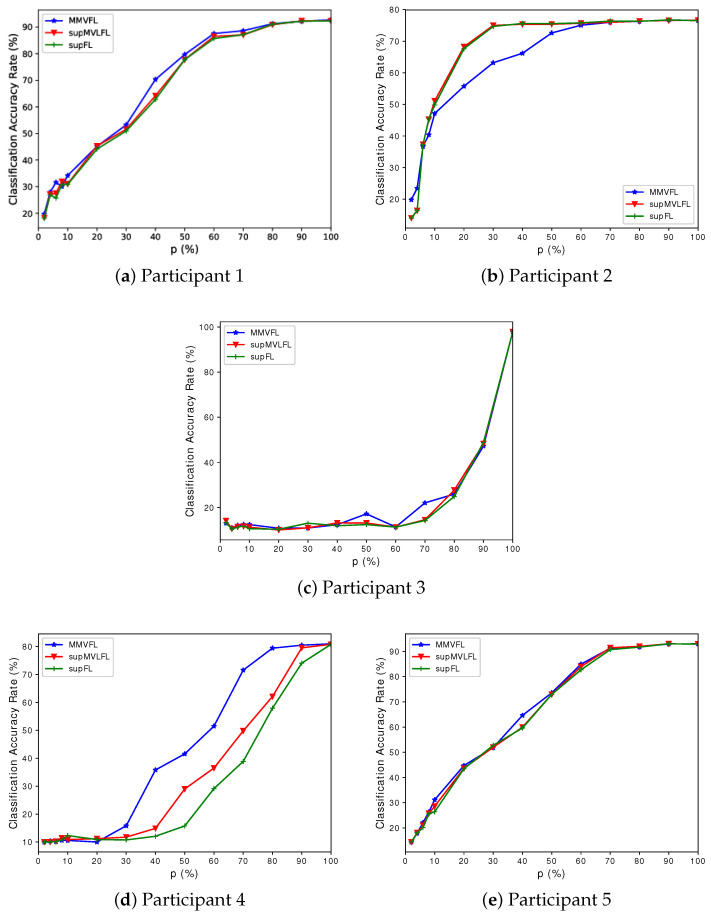
Performance of MMVFL and competing algorithms on *Handwritten* in classification as a function of the percentage of features selected *p* (%).

**Figure 4:**
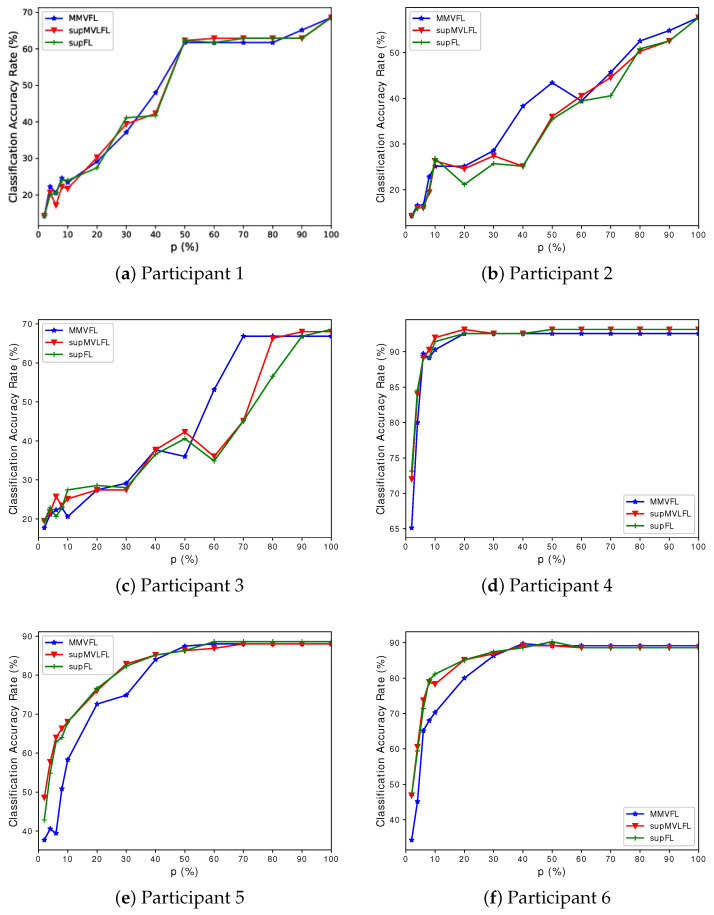
Performance of MMVFL and competing algorithms on *Caltech7* in classification as a function of the percentage of features selected *p* (%).

**Figure 5:**
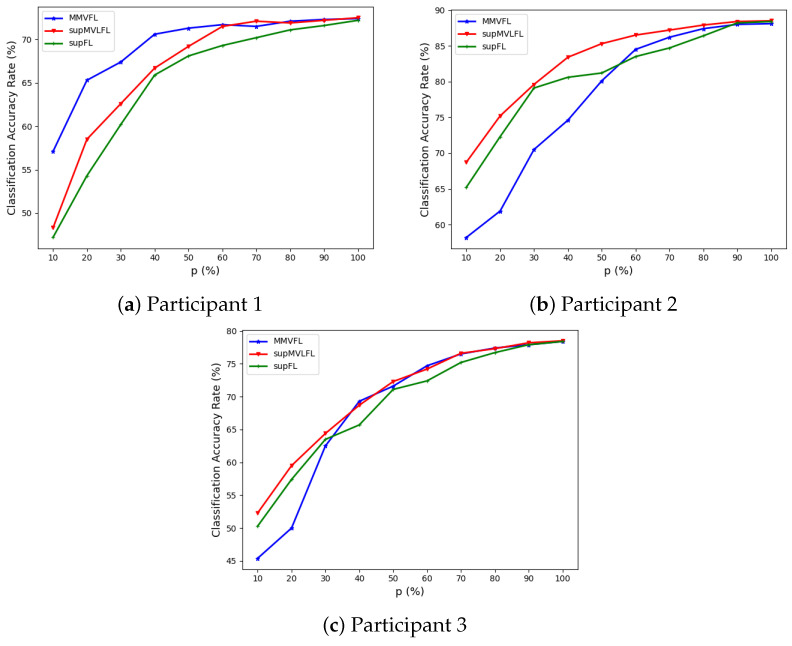
Performance of MMVFL and competing algorithms on *Isolet* in classification as a function of the percentage of features selected *p* (%).

**Figure 6:**
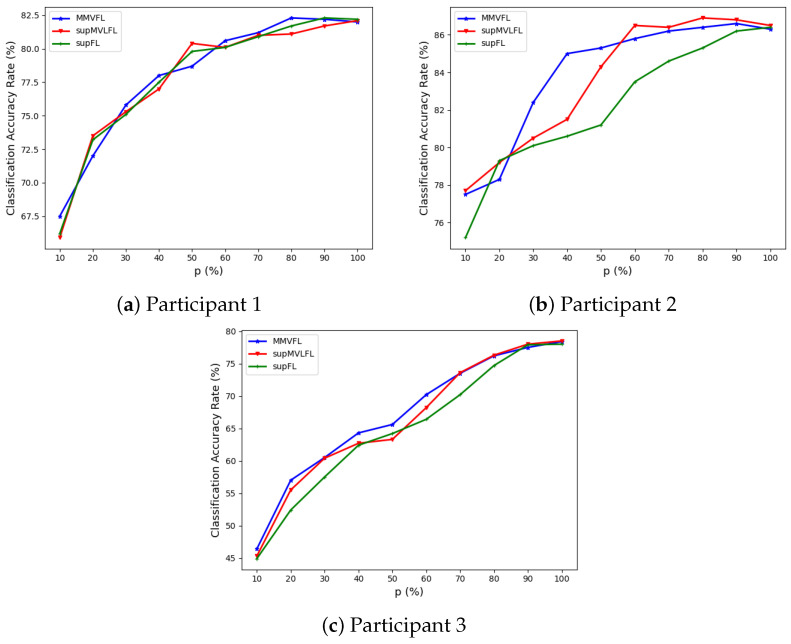
Performance of MMVFL and competing algorithms on *Relathe* in classification as a function of the percentage of features selected *p* (%).

We present the classification results of MMVFL and the comparison algorithms on the four datasets in Figure 3, Figure 4, Figure 5 and Figure 6. The results of the classification performance provided by MMVFL being comparable with the two competitors demonstrate that it is able to effectively share label information from the label owner participant to other participants under VFL settings to train a global FL model. As a side note, the comparison between supFL and supMVLFL shows that MVL helps improve learning performance in this experiment. Meanwhile, in some cases, MMVFL can achieve comparable or even better performance using a smaller number of important features than other approaches using all the features. As discussed in Section 4, by discarding features that are less important to the FL system based on the feature importance evaluation scheme proposed in Section 3.2, the resources required, such as communication bandwidth, computing devices, and memory space, can be reduced. This is especially advantageous for VFL systems under incremental learning settings.

## 6. Conclusions and Future Work

In this paper, we proposed a multi-participant multi-class vertical federated learning (MMVFL) framework, which shares the label information from its owner to all the other participants without data leakage. Unlike similar existing techniques that can only support two participants, MMVFL can work in more complex scenarios, making it suited for a wider range of applications. To the best of our knowledge, this is the first attempt to transfer a multi-view learning approach into the VFL setting. Experimental results on feature selection demonstrate that the performance of MMVFL can achieve comparable performance to its supervised counterparts.

In subsequent research, we will focus on four major directions to further enhance MMVFL. Firstly, we plan to explore how to incorporate more sophisticated classification techniques into this framework to expand its applicability. Secondly, we will embark on exploring the combination of MVL with HFL. Thirdly, we will explore the effect of relationships across tasks among different participants in VFL on the overall FL model performance. Lastly, we will improve MMVFL in aspects such as communication efficiency and data security protection.

## Figures and Tables

**Table 1:** Table of notations.

Notation	Definition
A	Matrix
${∥\cdot ∥}_{2,1}$	$l_{2,1}$-norm
X	Dataset
*K*	Number of participants
$N_{c}$	Number of classes
*N*	Number of samples
$d_{k}$	Data dimensionality at client *k*

**Table 2:** Properties of the datasets.

	Handwritten	Caltech7	Isolet	Relathe
Data dimensionalities of all views	240, 76, 216, 47, 64	48, 40, 254, 1984, 912, 528	200, 200, 217	1400, 1400, 1522
Training samples/class	120	20	40	400
Validation samples/class	40	5	20	200
Number of classes	10	7	26	2

## Data Availability

Data are contained within the article.
